# Structure-Based Analysis of A19D, a Variant of Transthyretin Involved in Familial Amyloid Cardiomyopathy

**DOI:** 10.1371/journal.pone.0082484

**Published:** 2013-12-17

**Authors:** Priscila Ferreira, Oliveira Sant’Anna, Nathalia Varejão, Cinthia Lima, Shenia Novis, Renata V. Barbosa, Concy M. Caldeira, Franklin D. Rumjanek, Salvador Ventura, Marcia W. Cruz, Debora Foguel

**Affiliations:** 1 Instituto de Bioquímica Médica, Universidade Federal do Rio de Janeiro, Rio de Janeiro, Rio de Janeiro, Brazil; 2 SONDA, Universidade Federal do Rio de Janeiro, Rio de Janeiro, Brazil; 3 Centro de Estudos de Paramiloidose Antônio Rodrigues de Mello, Hospital Universitário Clementino Fraga Filho, Universidade Federal do Rio de Janeiro, Rio de Janeiro, Rio de Janeiro, Brazil; 4 Institut de Biotecnologia i Biomedicina and Departament de Bioquímica i Biologia Molecular, Universitat Autònoma de Barcelona, Bellaterra, Spain; University of Pittsburgh School of Medicine, United States of America

## Abstract

Transthyretin (TTR) is a tetrameric beta-sheet-rich protein. Its deposits have been implicated in four different amyloid diseases. Although aggregation of the wild-type sequence is responsible for the senile form of the disease, more than one hundred variants have been described thus far, most of which confer a more amyloidogenic character to TTR, mainly because they compromise the stability of the protein in relation to monomer formation, which upon misfolding is intrinsically aggregation-prone. We report the case of a Brazilian patient suffering from a severe cardiomyopathy who carries a rare mutation in exon 2 of the TTR gene that results in an Ala to Asp substitution at position 19 (A19D). The putative pathogenic mechanisms of this variant were analyzed *in silico*. We constructed a structural model for the A19D tetramer from which its thermodynamic stability was compared to that displayed by the V30M (more amyloidogenic than WT-TTR) and T119M (non-amyloidogenic) variants. The FoldX force field predicted that A19D and V30M are 10.88 and 8.07 kCal/mol less stable than the WT-TTR, while T119M is 5.15 kCal/mol more stable, which is consistent with the aggregation propensities exhibited by these variants. We analyzed the step in which the tetramer-dimer-monomer-unfolded monomer equilibrium might contribute the most to the increased or decreased amyloidogenicity in each variant. Our results suggest that the concentration of four non-native negative charges occur inside thyroxine-binding channels, and the loss of contacts at both the tetrameric and dimeric interfaces would account for an overall decreased stability of the tetramer and the consequent enhanced amyloidogenicity of the A19D variant. As far as we know, this is the first description of a non-V30M mutation in Brazil.

## Introduction

Transthyretin (TTR) is secreted by the liver, choroid plexus and retinal cells and is a homotetrameric protein that transports thyroxine (T4) and retinol. In recent years, many studies have been performed on TTR to address its involvement in several amyloid diseases such as senile systemic amyloidosis (SSA), familial amyloid polyneuropathy (FAP), familial amyloid cardiomyopathy (FAC) and central nervous system amyloidosis (CNSA). SSA is caused by the aggregation of wild-type protein (WT-TTR), primarily in the heart, and more than 100 different point mutations are associated with the other three TTR-related amyloidoses [1,2]. 

FAP type I is the most common of the TTR-associated amyloidoses. This disease causes a wide spectrum of symptoms mainly involving neuropathy [3,4]. V30M is the most common FAP-associated mutation worldwide and it is endemic in Portugal and Japan. Until now, this mutation has been the only one described in Brazil [5,6]. Position 30 is located on exon 2, and it is a hot spot because four different substitutions at this position have been described [7–9]. Other hot spots in this exon are the codons for amino acids 18, 33, 42, 45 and 47 (http://amyloidosismutations.com/). All these hot spots lead to cardiomyopathies exclusively or associated with a peripheral neuropathy component. Exon 3 of TTR contains the greatest number of mutations that lead to FAC development such as Thr60Ala and Ile68Leu, although Val122Ile is the most common mutation associated with FAC, which is found on exon 4 [10–12]. 

**Figure 1:**
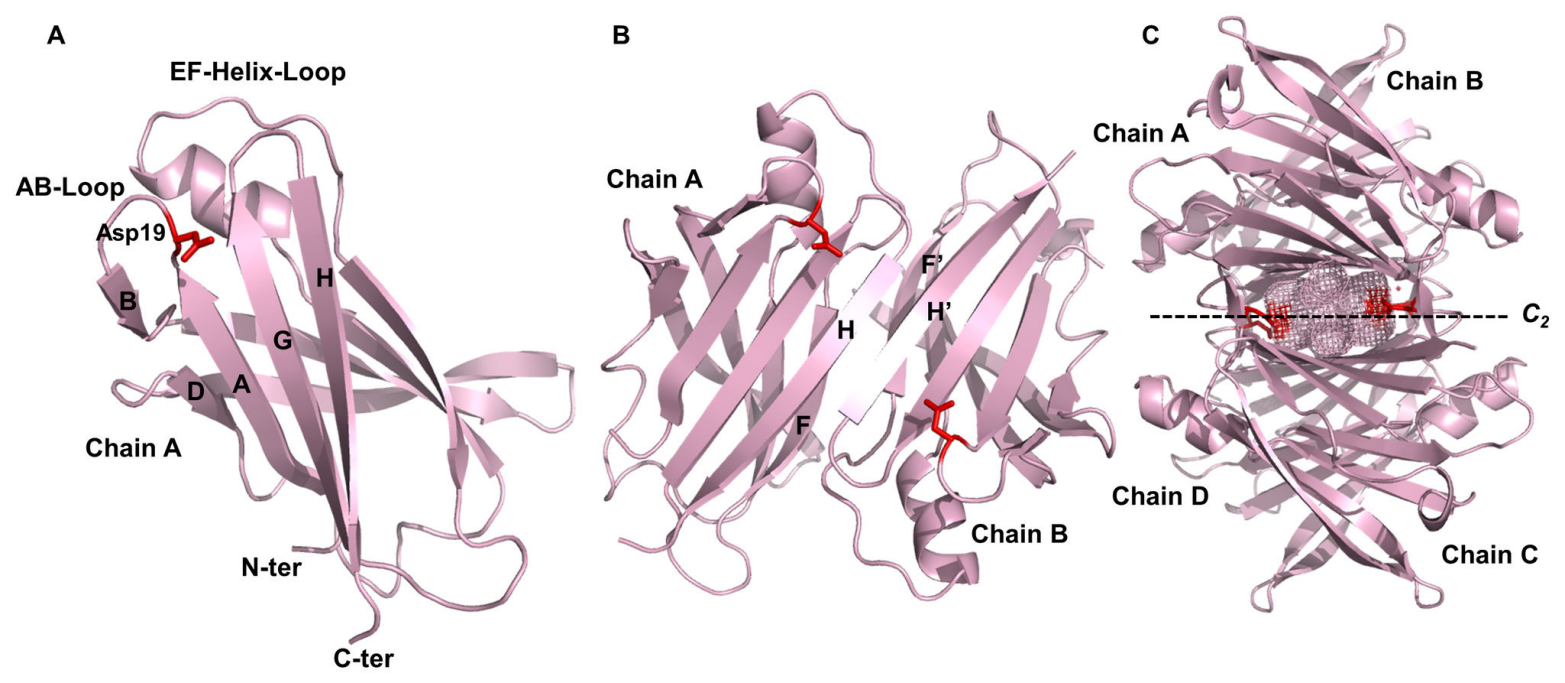
Spatial orientation of Asp19 (red sticks) on TTR monomer (A), dimer (B) and tetramer (C). Position 19 is located at the AB-loop (**A** and **B**). The Asp side chain extends across the thyroxine binding channels at the center of the tetramer (**C**). The structural models of A19D were generated by FoldX using a 1F41 PDB WT-TTR structure. Images were drawn with PyMOL.

TTR is a beta-sheet-rich tetrameric protein, as revealed by X-ray crystallography [13–15]. Each monomer (A, B, C and D) is composed of two four-stranded β-sheets (DAGH and CBEF), which are connected by loops with a short α-helix located between β-strands E and F (Figure 1A). Dimer AB and structurally symmetric dimer CD are held together by hydrogen bonds and other non-bonded contacts between β-strands H and H’, and between F and F’, where (’) stands for a different monomer (Figure 1B). Finally, dimers AB and CD associate mainly by hydrophobic interactions between loops AB and A’B’ and GH with G’H’ resulting in the formation of a TTR tetramer (Figure 1C). Each tetramer possesses two T4 binding sites at the interface between dimers AB and CD (Figure 1C, dotted line).

It has been proposed that TTR aggregation starts by tetramer dissociation followed by misfolding of individual monomers [16–19]. Thus, the thermodynamic stability of a given variant seems to be directly connected to its amyloidogenic potential [20–23]. 

Here we describe a heterozygous mutation (Ala19Asp, A19D) in exon 2 of the TTR gene from a Brazilian patient whose family has Swedish-German origins. This mutation caused a severe cardiac impairment as the main symptom, accompanied by neuropathic pain, leading to the inclusion of A19D as a new FAC-related variant. Position 19 is located within the AB-loop, a region that is directly involved in the dimer-dimer contacts (i.e. A/C-B/D) that lead to tetramer formation (Figures 1A and C). This interface bisected by the crystallographic C2 axis is the most unstable of the dimer-dimer interfaces [19]. AB loops also face the T4 binding channels, which compelled us to evaluate the structural consequences of the presence of four negative charges from aspartic acid on the architecture of these channels [24–26].

After finding this new variant in the Brazilian population, we investigated its influence on tetramer thermodynamic stability by using the bioinformatics tools FoldX and PDBSum. Besides the WT-TTR, two other well-characterized variants of TTR were used as controls, namely, V30M (amyloidogenic) and T119M (non-amyloidogenic). Interestingly, FoldX predicted that the tetramer with A19D has a decreased overall thermodynamic stability when compared to WT-TTR and to V30M, an unstable variant of TTR. FoldX also provided an assessment of the ΔΔG (in relation to the WT-TTR) for each step of TTR unfolding (tetramerdimermonomerunfolded monomer). In accordance with available experimental data, the V30M mutation compromises the stability of the monomer, whereas the increased stability of T119M results from a reduction in the tetramer dissociation. On the contrary, the dissociation of tetramers into dimers is favored in the case of A19D compared with WT-TTR. Additional A19D model structural analyses allowed us to map all the non-covalent contacts (non-bonded and H-bonds) perturbed by the A19D mutation. The present study illustrates the use of bioinformatic tools as a primary approach for correlating protein stability, amyloid propensity and hopefully, disease progression in TTR-linked diseases. 

## Materials and Methods

### Ethics Statement

 This study presents data on signs and symptoms, neurological and cardiac assessments, biomarkers and quality of life of one patient that was referred to CEPARM and received approval from the Ethics Committee of University Hospital from the Federal University of Rio de Janeiro, Brazil for clinical evaluation. Written informed consent was obtained from the patient prior to participation in the study and all procedure were conducted in accordance with de Declaration of Helsinki.

### Patient Privacy and Informed Consent for Publication

 The patient in this manuscript has given written informed consent, as outlined in the PLOS consent form, to publication of his case details. 

### Enzymatic Amplification of Genomic DNA

 Total DNA was isolated from peripheral leukocytes. The samples were extracted using a phenol-chloroform protocol. Exons 2, 3 and 4 of the TTR gene were amplified with 1 μL of DNA and the appropriate set of oligonucleotide pair primers as previously described [27]. Amplification was performed with an AmpliTherm DNA thermal cycler. The thirty cycles consisted of denaturing at 94°C for 1 min, annealing at 60 °C for 1 min, and extending at 72 °C for 1 min. The PCR product was purified by Exo-Sap with 5 μL of PCR product, 1 μL of exonuclease I and 0.5 μL of Shrimp Alkaline Phosphatase. The samples were then incubated for 5 min at 37°C for the enzymatic reaction and the enzymes were inactivated by incubating the sample at 65 °C for 15 min.

### Cloning in the pGEM-T vector

 Twenty-five μL of PCR product was purified with the Exo-Sap protocol. Four μL of purified PCR product was added to a solution containing 1 μL of plasmid vector pGEM-T, 5 μL of 2x ligation buffer, 1 μL of T4-DNA ligase, 9 μL of de-ionized water and then incubated overnight at 4 °C. The preparation was used to transform the DH5ɑ E. coli strain. PCR was carried out with one part of the colonies to confirm the presence of exon 2 and then analyzed by agarose gel electrophoresis with examination under UV light. The rest of the colony was grown in LB medium and the plasmid was extracted using a plasmid extraction kit (Promega Inc.) and then sequenced.

### Direct DNA sequencing of TTR exons

The amplification products were sequenced by Big Dye Terminator Cycle Sequencing method with the same primers that were used for PCR amplification in an ABI (3130) Applied Biosystems sequencer. Sequence traces were automatically compared with the normal TTR reference sequence NG_009490.1. The resulting sequences were analyzed by Vector NTI Advance 10 Software (Invitrogen).

### FoldX prediction

We built three different models (A19D, V30M and T119M) by using FoldX (http://foldx.crg.es/) command *Buildmodel*  with the original WT-TTR structure as deposited in the PDB under code 1F41; each model was generated with five runs, and they converged well. The resulting structures were used to calculate the ΔΔGs values presented in the Results section. The commands *Analyze Complex* and *Stability* were used to calculate the ΔΔGs for interface dissociation and unfolding, respectively. The energies are an automatic output in FoldX, and the changes in native stability upon mutation were estimated as the difference between the energy of the wild type protein and that of the mutant protein (ΔΔG = ΔG_mut_ - ΔG_wt_). ΔΔGs values above 1.6 kcal/mol should significantly affect variant stability, because they correspond to twice the intrinsic standard deviation of FoldX [28]. 

## Results

### Description of a new TTR variant in Brazil encoding an alanine to aspartic acid substitution at position 19 (A19D)

With a goal of establishing a diagnostic center for TTR-related amyloidoses, our groups have established the protocols for sequencing TTR genes and offer this service to the population of the State of Rio de Janeiro and to the Brazilian people in general. 

**Figure 2:**
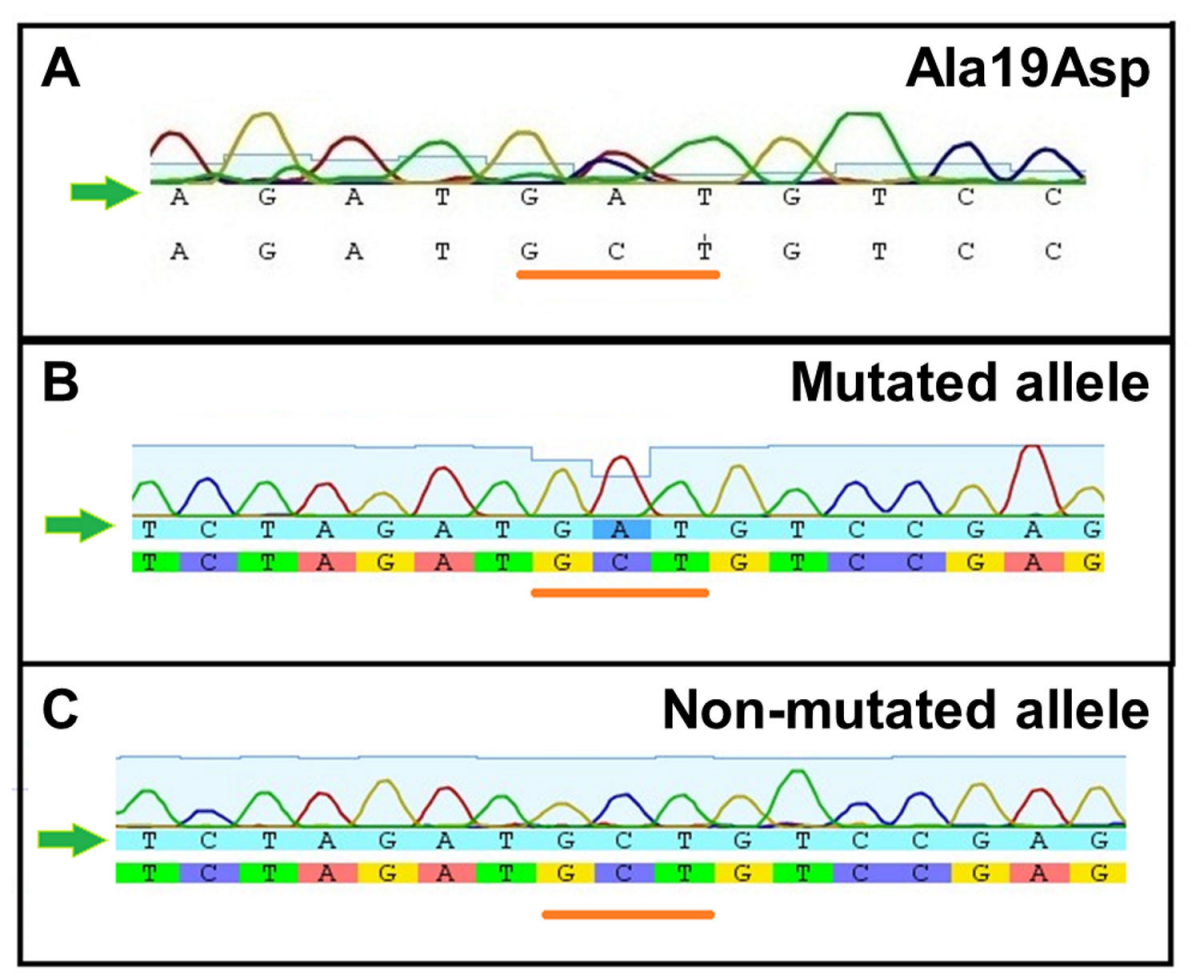
TTR Exon 2 from a Brazilian patient with cardiomyopathy carries a mutation at position 19 (A19D). Sequencing electropherogram of exon 2 of the TTR gene shows a heterozygous C/A mutation at position 47 (A). A sequence analysis of individual clones shows one that carries the mutated (**B**) and non-mutated alleles (**C**). The orange underlines indicate the mutation site (cytosine for adenine).

The TTR gene sequence (at exons 2, 3 and 4) from a Brazilian patient with a severe cardiac impairment showed a single and unusual base substitution in exon 2 at position 117 (GCT to GAT), indicating the replacement of an alanine by an aspartic acid at position 19 (A19D; Figure 2A, underlay in orange). It is possible to note the heteroplasmy present in the electropherogram of the TTR gene in this patient, indicating heterozygosity for this mutation inherited from his father, who also presented FAC symptoms. No other mutation was found in the rest of exon 2 or in exons 3 or 4 of TTR (data not shown).

To confirm the presence of this rare mutation, the PCR products were cloned and sequenced to separately analyze the alleles. Figures 2B and C confirm the presence of two distinct clones, one related to the mutant (panel B) and the other to the WT allele (panel C). 

As far we know, this is the first description of a patient with the A19D mutation in Brazil. 

While we could not find this mutation in the data bank (http://amyloidosismutations.com/), a recent report by Schönland et al. (2012) reported this TTR variant in a German patient with cardiac amyloidosis [29]. Interestingly, the patient we found in Brazil is of Sweden-German origin, originally from the State of Santa Catarina (south Brazil), which is a state with a large Germanic community. 

### Case report of one Brazilian FAP patient with a rare mutation on the TTR gene

The patient was first seen at the University Hospital of the Federal University of Rio de Janeiro, at CEPARM (Centro de Estudos de Paramiloidose Antônio Rodrigues de Mello; http://ceparm.com) on March 2012 with a history of sensory complaints in the extremities since 2009 as characterized by neuropathic pain, numbness, tingling and balance abnormality. His walking disability was graded as I (sensory disturbances in his feet but able to walk without difficulty) that evolved seven months later to grade II (some difficulties with walking but can walk unaided). He also presented with dyshidrosis, dry eyes and erectile dysfunction since the previous year.

His cardiac complaints consisted of palpitations, dizziness and dyspnea with heart failure severity classified by the NIHA as IV (cardiac limitations with any physical activity). Other manifestations included early satiety, nausea and constipation since the previous year. The neurological examination showed an absence of thermal and pain sensation on both upper and lower limbs with decreased tactile sensation in the four limbs and vibratory sensation in the lower limbs. The aquilean reflex was not noted and muscle strength was normal in both distal and proximal aspects of the four limbs. The electroneuromyography study revealed the absence of a sympathetic skin response in the foot and a few abnormalities in nerve conduction, with reduced compound muscle action potential amplitude in the left peroneal nerve, and no observed sural nerve compound nerve action potential. A salivary gland biopsy demonstrated the presence of amyloid deposits in this patient. 

The patient had an elevated NT-BNP level (258 pg/mL), which is consistent with cardiac dysfunction. The ECG detected a first degree atrial ventricular block and Holter analysis revealed a nonsustained ventricular tachycardia. The echocardiogram revealed a left ventricular hypertrophy with a granular appearance, more evident in the septum, a preserved left ventricular systolic function and grade III diastolic dysfunction (restrictive pattern, left ventriculum ejection fraction of 48%). There was a moderate left atrial enlargement and mild mitral regurgitation. Mild pulmonary hypertension was also noted as well as minimal tricuspid and pulmonary regurgitation. An MRI confirmed the presence of ongoing atrial dysfunction. To ameliorate the cardiac failure, the patient was treated with amiodarone, ACE inhibitors, diuretics and beta blockers. This patient has a brother with the same mutation, who underwent cardiac transplantation as a consequence of severely compromised cardiac function. A liver and heart transplant intervention at a referral center is being evaluated for the present patient. 

### Structural and thermodynamic stability prediction of the alanine to aspartic acid substitution at TTR position 19

Next, we attempted to dissect the possible TTR structural changes caused by Ala19Asp replacement and how it could account for differences in the thermodynamic stability and amyloidogenicity of this new variant by using different bioinformatics tools. The energetics of protein folding and association (ΔG_f_ and ΔG_a_) are dictated by the internalization of hydrophobic groups, formation of optimal non-covalent electrostatic interactions (enthalpy) and maximization of conformational entropy [30]. 

FoldX is a well-validated algorithm used for several purposes such as generating structural models based on tridimensional high resolution structures, predicting the impact of point mutations on protein stability [31,32], and searching for ion-binding sites on the primary protein sequence [33], among other possibilities. Because FoldX is an all-atom force field analysis, it can dissect the contribution of all inter and intra-chain interactions responsible for protein structural stability, which include van der Waals contacts, main and side chain H-bonds and other weak interactions, and the energy associated with polar and non-polar residue solvation [31]. 

We initially used FoldX to create a structural model for A19D by using the crystallographic structure of the WT-TTR (PDB: 1F41). As seen in many (but not all) variants of TTR for which the structures have been solved by X-ray crystallography [14,15], no global changes were observed when the A19D structure was superimposed over that of the WT-TTR protein (Figure S1). From this model, the energies associated with all interactions that are formed or lost upon mutation were quantified and used to calculate the predicted change in native thermodynamic stability (ΔΔG_total_= ΔG_mut_ - ΔG_wt_). FoldX also analyzes the contribution of subunit binding energies within the tetrameric structure (monomer-monomer and dimer-dimer interactions) and thus describes the energetics associated with a dissociation into subunits as well as unfolding. In the case of TTR, the sequences of events are as follows: step 1, dissociation of the tetramer into dimers (ΔΔG_d1_); step 2, dissociation of the dimers into monomers (ΔΔG_d2_); and step 3, monomer unfolding (ΔΔG_u_). We presumed that the TTR tetramers are converted into AB and CB dimers in the first step because, as shown before by Foss and coworkers, the AB/CD interface seems to be the weakest one in the tetramer [16,19,34]. 

It is important to mention that ΔΔG_total_ represents the difference in free energy from the final (unfolded monomers) to the initial state (native tetramer) of the mutant in comparison to the same difference from the WT-TTR, and cannot be mathematically calculated assuming an additive contribution of ΔΔG_d1_, ΔΔG_d2_ and ΔΔG_u_ terms to the total unfolding energy.

To validate the accuracy of FoldX in predicting the energetics of A19D, we selected two well-studied variants of TTR, namely V30M, which is amyloidogenic, and T119M, a non-amyloidogenic variant. The complete sequence of commands and parameters used to perform the analyses are described in the Material and Methods section (see also Schymkowitz et al., 2005 for more details).

The resulting ΔΔG_total_ values were +10.88, +8.07 and -5.15 kcal/mol for A19D, V30M and T119M, respectively (decreased and enhanced stabilities furnish positive and negative values, respectively; Table 1). Because the A19D mutation was found in a FAC patient, we did expect that FoldX would attribute a positive ΔΔG_total_ to this new variant; however, to our surprise, A19D was predicted to be even more unstable than the most frequent FAP-associated variant, namely V30M, which is 2.81 kcal/mol more stable than A19D.

**Table 1:** Predicting the stabilities of TTR variants in relation to the WT-TTR by FoldX.

	**A19D-TTR**	**V30M-TTR**	**T119M-TTR**
**ΔΔG_tot_**	10.88	8.07	- 5.15
**ΔΔG_d1_**	4.65	0.00	- 5.24
**ΔΔG_d2_**	0.27	0.01	0.57
**ΔΔG_unf_**	1.24	3.03	1.02

FoldX generated structural models of the variants A19D, V30M and T119M. **ΔΔ**G values were calculated (kcal/mol), in which ΔΔG = ΔG_mut_ - ΔG_wt_. These values were used to probe the stability of the mutant in relation to the WT-TTR. Positive and negative values (standard deviation of 0.8 kcal/mol) indicated a decrease or increase in protein stability, respectively. **ΔΔ**G_total_ is related to the disruption of all intra- and intermolecular contacts in the tetramer. **ΔΔ**G_d1_ and **ΔΔ**G_d2_ are related to tetramer dissociation into dimers and dimers into monomers, respectively. **ΔΔ**G_unf_ describes the energetics associated with the unfolding of the separate monomers.

Next, we used FoldX to dissect the impact of A19D, V30M and T119M mutations on each step of TTR denaturation (ΔΔG_d1,_ ΔΔG_d2_ and ΔΔG_u_). The ΔΔG_d1_ value for highly stable, non-amyloidogenic TTR variant T119M was negative and equal to -5.24 kcal/mol, which means there was a higher energetic barrier for dissociation (formation of dimers AB and CD) in comparison to the WT-TTR, and its ΔΔG_d2_ (dissociation of AB and CD dimers into monomers) was virtually identical to the WT-TTR (+0.57 kcal/mol). The unfolding step (ΔΔG_u_) suggests that T119M monomers are indeed slightly less stable than WT-TTR monomers (Table 1). 

For the amyloidogenic variant V30M, FoldX did not predict differences in any of the dissociation steps in relation to the WT-TTR (ΔΔG_d1_ and ΔΔG_d2_ values were equal to zero), suggesting that the inter-monomer interactions responsible for maintaining the dimers and tetramer of V30M were not affected by the Val-Met replacement at position 30. As for the unfolding step, the ΔΔG_u_ for V30M was positive and high (+3.03 kcal/mol), suggesting that the overall instability of this variant and its enhanced amyloidogenicity is caused by a mutation-induced destabilization of intra-chain contacts (Table 1).

Interestingly, a different scenario was observed for A19D in relation to V30M, in which the first dissociation step presented a large and positive ΔΔG_d1_ of +4.65 kcal/mol, suggesting that Ala substitution by Asp at position 19 destabilizes the inter-chain contacts that held the tetramer together (A/C-B/D). Again, no stability difference in dimers AB and CD were observed in relation to the WT-TTR, and A19D monomers were shown to be less stable than the WT-TTR monomers (Table 1; ΔΔG_u_ = +1.24). 

Taken together, the predicted thermodynamic stabilities calculated by FoldX suggest that the tetramers composed of V30M and A19D are destabilized in relation to the WT-TTR; the instability of the former resides in the intra chain contacts that held the monomers folded, while in A19D, the contacts between dimers AB and CD (A/C-B/D, *C*_*2*_ axis) seem to be compromised by the mutation. 

### Evaluating the structural modifications caused by Ala19Asp substitution and their implication for predicting decreased thermodynamic stability

Although FoldX does not rearrange Cɑ upon mutation, the crystallography structures of many TTR mutants solved to date show no significant backbone deviation. Therefore, we decided to approach possible structural side chain rearrangements caused by the A19D mutation that could explain its decreased stability by using Foldx.

**Figure 3:**
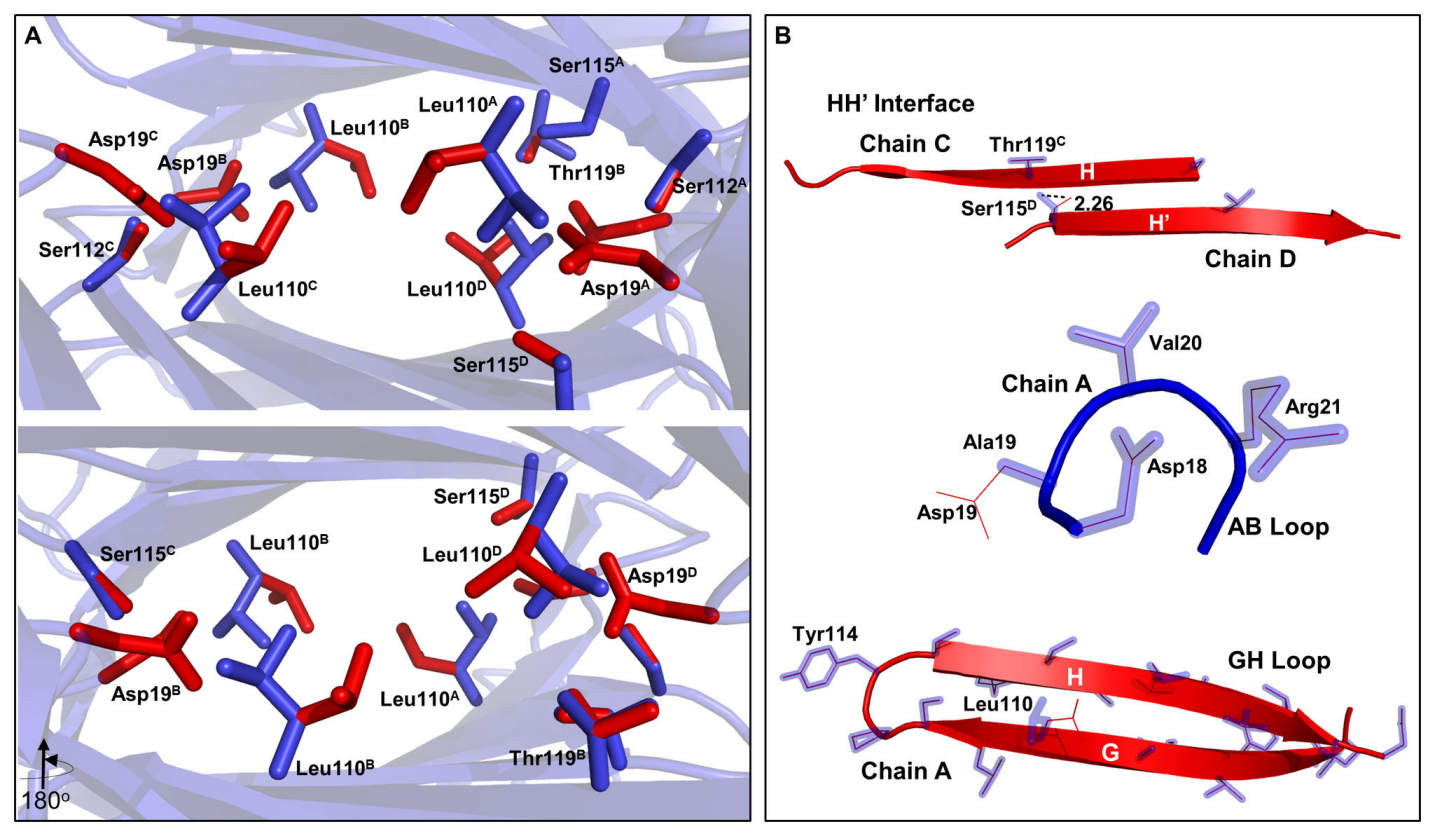
Changes in the orientation of amino acids in the A19D mutant of transthyretin. The WT-TTR tetramer (PDB ID: 1F41 after symmetry operation) and A19D (generated by FoldX) were superimposed. (**A**) A glimpse in the thyroxine binding channel shows the residues that change their orientation after the insertion of four Asp residues at position 19. They are Asp19, Ser112 and 115, Leu110 and Thr119. The letters associated with the residues denotes the chain to which they belong. The residues in WT and A19D are colored in blue and red sticks, respectively. (**B**) Highlight of the regions involved in dimerization (upper) and tetramerization (middle and lower) of WT (blue) and A19D (red). The structures were aligned by TopMatch and the images were drawn by PyMOL.

Position 19 is located on the AB-loop (Figures 1A and 3B, middle), a small region (five amino acid residues long, position 18-22) that connects β-strand A and B, which is involved in TTR tetramerization. Other destabilizing variants of the TTR map into this region, such as D18E and V20I, both of which cause cardiac effects [2,35]. Interestingly, urea denaturation studies performed with V20I showed that although this tetramer was less stable than the WT-TTR, its monomer was equally stable as its wild type counterpart [36]. The destabilizing effect predicted by FoldX at position 19 in combination with the study performed by Jenne and coworkers suggests that AB-loop mutations tend to decrease the stability of TTR tetramers with much less impact on monomer stability.

It is also important to note that the four AB loops of the tetramer face the T4 binding channels (Figure 1C, 3A). Thus, we expect that the bulky side chains of the four aspartic acid residues might compromise the architecture and binding properties of these channels. Figure 3A shows the T4 binding channels, highlighting the residues that displayed major structural rearrangements as a consequence of the mutation, namely Leu110, Ser112, Ser115 and Thr119. Changes in side chain distances within A19D are presented in Table 2. Note that they are positioned in regions that are involved in TTR dimer and tetramer assembly (Figure 3B).

**Table 2:** Changes in side chain distances of the residues that underwent spatial reorientation as a consequence of Ala19Asp substitution in the TTR structural model.

	**Leu110**	**Ser112**	**Ser115**	**Thr119**
	**(G-Strand)**	**(GH-Loop)**	**(GH-Loop)**	**(H-Strand)**
**Chains**	C^δ1^/C^δ2^	O^γ^	O^γ^	O^γ1^/C^γ2^
**A**	(3.60)/(3.85)	(0.14)	(0.19)	(-----)/(-----)
**B**	(3.77)/(3.61)	(-----)	(-----)	(0.20)/(0.21)
**C**	(3.19)/(3.72)	(0.32)	(-----)	(-----)/(-----)
**D**	(3.44)/(2.85)	(-----)	(2.26)	(-----)/(-----)

All distances are in Angstroms. Traces (----) indicate that there were no distance changes in this specific residue. FoldX generated WT and A19D TTR models that were superimposed in TopMatch and calculations were performed by measuring the distances from the side chain atoms of the variant in relation to the same residue in WT-TTR.

Moreover, the seminal work by Blake and coworkers on the crystal structure of WT-TTR in complex with T4 allowed for the characterization of three halogen binding pockets (HBP 1-3) in each hormone-binding site located at the A/C-B/D interface of the tetramer [24–26]. Inner pockets HBP3 and HBP3’ are formed by the side chains of residues Ala108, Leu110, Ser117 and Thr119; HBP 2 and 2’ are formed by Leu17, Ala108, Ala109 and Leu110 and HBP1 and HBP1’, which are the outer pockets, are formed by Met13, Lys15, Leu17, Thr106, Ala108 and Val121. Among all the residues involved in HBP formation, Leu110 (from all monomers), Ser112 (from chains A and C), Ser115 (from chains A and D) and Thr119 (from chain B) underwent spatial reorientation in A19D (Table 2), although the magnitude of the changes were much more prominent for Leu110 (all chains) and Ser115 (only in chain D). According to the crystal structure of complex TTR-T4, the amide nitrogen of Leu110 makes polar contacts with T4 and with the non-steroidal anti-inflammatory drugs that bind in these channels as well. Thr119 establishes hydrophobic and van der Waals contacts with the hormone inside the T4 channels [37]. Thus, we expect that the binding of T4 to TTR might be affected in this patient.

**Figure 4:**
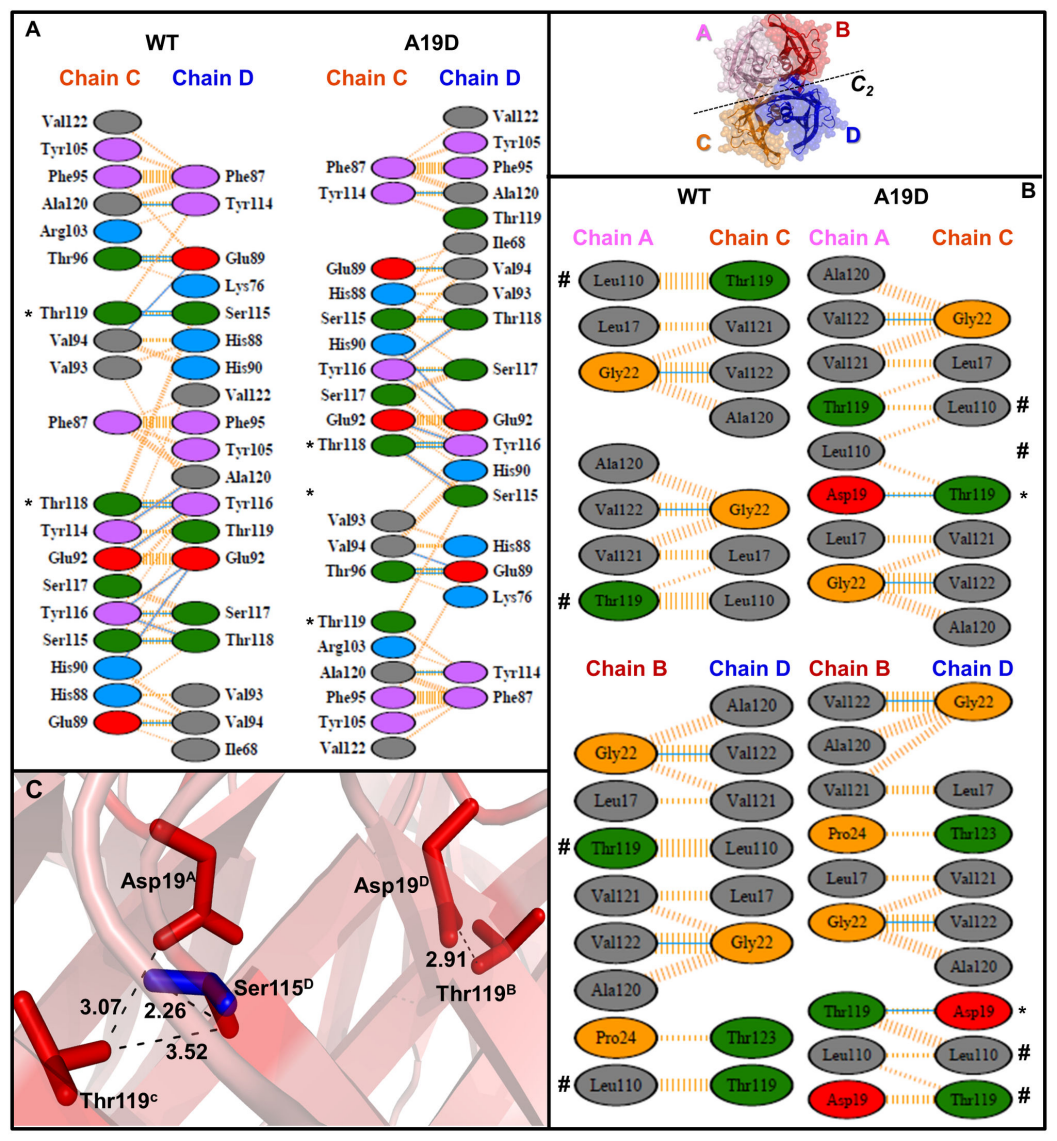
Alanine to aspartic acid substitution at position 19 affects the TTR dimer and tetramer interfaces. Overview of residues involved in atomic contacts at (**A**) dimeric and (**B**) tetrameric interfaces. The upper most panel depicts the tetrameric assembly of the four identical chains in A19D (A, B, C, and D). The hydrogen bonds are indicated by blue lines. Non-bonded contacts are shown in striped lines, in which the width is proportional to the number of atomic contacts. Asterisks and hashtags highlight changes in the H-bonds and non-bonded contacts within A19D, respectively. Amino acid residues are colored as neutral (green), aliphatic (gray), aromatic (purple), positive (blue), negative (red), and glycine (orange). Calculations were performed by the PDBSum database by using the tetramer models of WT-TTR and A19D generated by FoldX. (**C**) A closed view of the distances across the residues that changed orientation, causing the breakage of the two H-bonds between C/D interface (Thr119^C^ and Ser115^D^), and two new H-bonds are formed between chain A/C and B/D interfaces (Asp19^A^ and Thr119^C^, Asp19^D^ and Thr119^B^). Blue and red colors mark residue side chains in WT-TTR and A19D, respectively. The distance labels are denoted in Angstroms.

Next, to gain additional insights about the conformational differences underlying the decreased stability of A19D, we used the PDBSum package (www.ebi.ac.uk/pdbsum), which provides a dissection of the changes in intermolecular contacts between A/B, C/D, A/C and B/D interfaces (Figure 4, Table 3). Interestingly, the analysis of these interactions revealed an intricate profile with several gains and losses of non-covalent contacts (H-bonds and non-bonded contacts; Table 3). 

**Table 3:** Summary of important parameters involved in dimer and tetramer formation of the WT-TTR and A19D variant.

	**NB-contacts**	**Net gain/loss**	**NB-contacts**	**Net gain/loss**	**H-bonds**	**Net gain/loss**
	(overall)	(in A19D)	(Leu110-Thr119)	(in A19D)	(overall)	(in A19D)
**Dimeric**	**Interface**					
**A/B**	156/157	+1	---	---	18/18	0
**C/D**	159/157	-2	---	---	16/15	-1
**Tetrameric**	**Interface**					
**A/C**	32/28	-4	8/3	-5	2/3	+1
**B/D**	30/31	+1	7/5	-2	2/3	+1

NB = non-bonded contacts. Calculations were performed by the PDBSum server. There are no salt bridges with a cutoff of 4 Å. Procheck analysis shows that 92.5% of the residues in both structures are in the most favored regions and none are in the disallowed ones. the same is true of WT-TTR (PDB: 1F41).

With respect to the dimeric interface, there is one more and two less non-bonded contacts in the dimeric A/B and C/D interfaces (Table 3), respectively, which leads to a net loss of one non-bonded contact in the A19D tetramer. With regards to the H-bonds, the dimeric C/D interface of A19D loses two H-bonds between Ser115-Thr119 and gains a new contact between Thr118-Ser115 (as marked by asterisks in Figure 4A), which leads to a net loss of one H-bond (Table 3) between subunits C and D. The loss of these H-bonds were caused by the movement of Ser115 (O^γ^, located in strand H’) from monomer D by 2.26 Å away from its original position, causing its distancing from Thr119 (O^γ^, located in strand H) from monomer C (Table 2 and Figure 4C). Because there is no gain or loss in the number of H-bonds at the A/B interface, the net change in the number of H-bonds at the dimeric interface is the loss of one H-bond at the C/D interface, which also loses two non-bonded contacts and is most likely the weaker dimeric interface of A19D. 

At the tetrameric interface (A/C-B/D), Asp19 side-chains from monomers A and D project into the dimer-dimer interface attracting Thr119 from monomers C and B, respectively, leading to the formation of two new H-bonds between these two amino acids, one at interface A/C (3.07 Å) and the other at B/D (2.91 Å) (Figure 4B asterisks and C and Table 3). In relation to the non-bonded contacts, two pairs of amino acids called our attention to A19D, namely Asp19-Thr119 and Leu110-Thr119 (Table S1). In the case of the former pair, four new contacts were established between residues 19 and 119 at the A/C and B/D interfaces (+1 and +3, respectively). However, the Leu110-Thr119 pair loses seven non-bonded contacts in A19D (Table S1), five in A/C and two at the B/D interface (Table 3, Figure 4B hashtag). Considering that the A/C-B/D interface is the weakest of the TTR tetramers (*C*_*2*_ axis), the loss of these seven contacts might substantially affect the already weak tetrameric interface in the case of A19D. 

In support of this hypothesis, an analysis of the tetrameric interface of T119M, which is the very stable variant of TTR, showed a gain of eight non-bonded interactions between the Leu110-Met119 pair, in addition to the two new interactions between Leu17-M119 (+10 in total, Table S1). Moreover, no differences were found in the contacts of the V30M variant, which is a tetramer with stability equal to the WT-TTR (Table S1 and Table 1, ΔΔG_d1_). Interestingly, an analysis of all other non-bonded contacts located at the tetrameric interface of V30M, T119M and A19D failed to locate any other significant differences in relation to the WT-TTR, suggesting that the Leu110-Thr(Met)119 pair might be responsible for most of the tetramer's stability (Table S1). 

**Figure 5:**
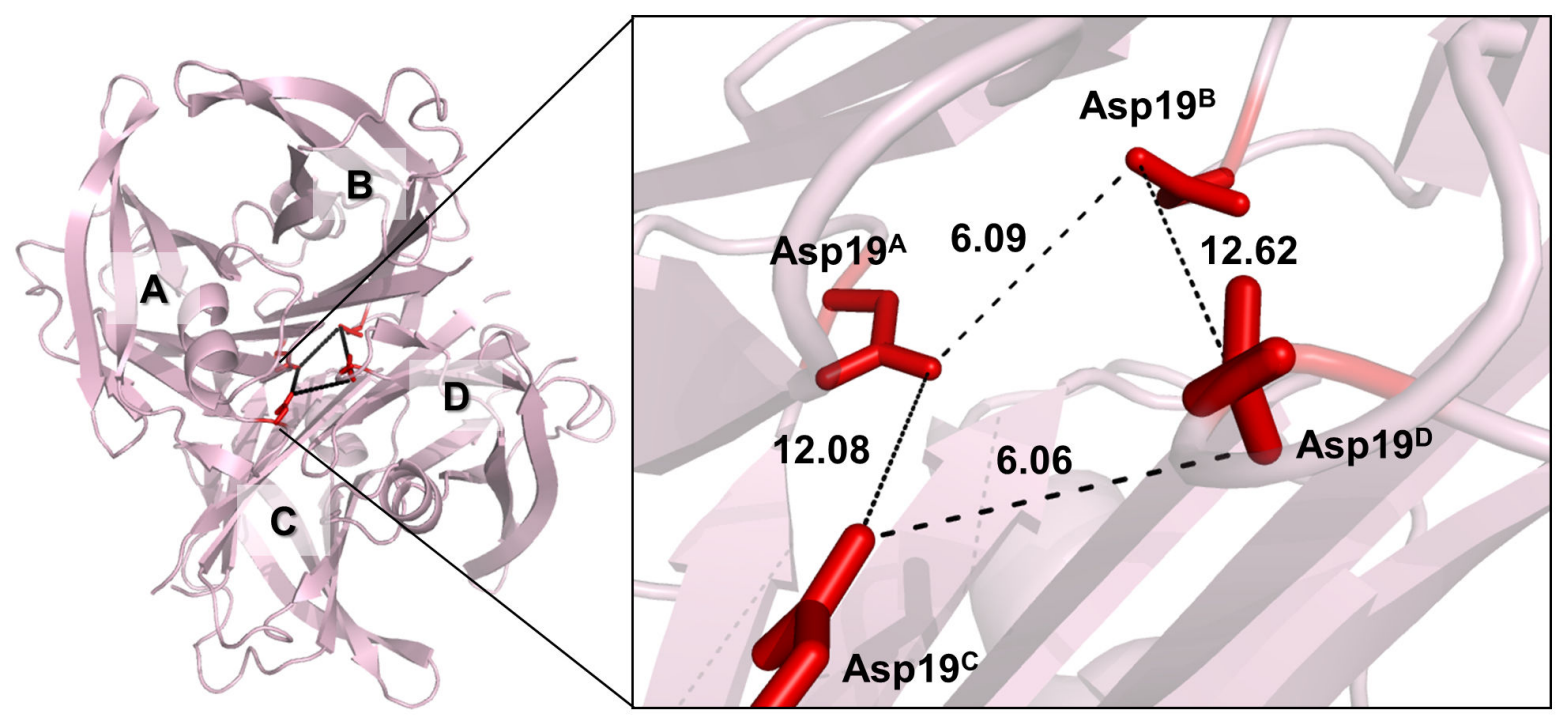
Insertion of an aspartic acid at position 19 causes an electrostatic clash within thyroxine binding channels. Dashed lines show the distances between the four Asp19 side chains inside the thyroxine binding channels. The O^d1^ and O^d2^ of Asp19 are colored in red. It is important to note that the O^d2^ from interfaces A/B and C/D are at distances of 6.09 and 6.06 Å. The image was drawn by PyMOL.

Because the AB loop has charged residues (Asp18 and Arg21), we analyzed the impact of the insertion of an extra negative residue on this loop (Asp19). Interestingly, Asp19 did not create or affect any intra or inter-chain ionic interactions (up to 6 Å) present in the TTR structure (ESBRI server, data not shown). However, as shown in Figure 5, the presence of four negatively charged groups from Asp 19 within the T4 channels might generate a repulsive electrostatic clash. In fact, the measured distances between Asp19 side chains across A/B and C/D interfaces measure 6.09 and 6.06 Å, respectively, which likely causes a charge repulsion contributing to the destabilization of A19D at the strongest interface (Figure 5). 

## Discussion

Until now, more than a hundred point mutations have been described in the TTR gene as the cause of three types of TTR-related amyloidoses (FAP, FAC and CNSA), which occur early in life. However, the WT-sequence of TTR responds during the senile form of the disease, which afflicts more than 10% of people older than 80. Thus, these mutations accelerate TTR amyloidogenesis with the accumulation of aggregated material in the target organ or tissue. 

There are nearly 200 X-ray structures of TTR including most of the variants thus far identified. None of them showed any major structural changes that could explain their enhanced amyloidogenicity [14,15]. Nonetheless, several biophysical studies have shown that there is an inverse correlation between the thermodynamic and kinetic stability of a given TTR variant and its amyloidogenicity [20–22,38]. This is true because the current model that explains TTR aggregation shows that the raw material for fibril formation is a partially unfolded monomer, which forms after tetramer dissociation [16–18,34,39]. Acidification (pH ~4) triggers this process and our group has shown that high hydrostatic pressure also elicits TTR aggregation at a pH close to 5.0 [21,40]. Thus, knowing the stability of a given TTR variant gives a strong indication of its aggregation propensity, which justifies the approach used in the present study, at least for a preliminary evaluation. 

Here we have described a new TTR variant in the Brazilian population, namely A19D, which is associated with severe cardiomyopathy. Until now, V30M has been the only variant of TTR reported in the Brazilian population, a variant that was inherited during Portuguese colonization. There is only one brief report of this variant in the world population [29], although it is not included in TTR databanks (http://www.ibmc.up.pt/mjsaraiva/ttrmut.html and http://amyloidosismutations.com/mut-attr.php). 

A19D thermodynamic stability was estimated here with FoldX and compared to that of V30M and T119M. FoldX allowed us to calculate the values of ΔΔG_total_, which showed that A19D and V30M are approximately 11 and 8 kcal/mol less stable that the WT-TTR, and T119M is 5 kcal/mol more stable than the WT-TTR. Thus, the forcefield precisely predicted the expected stability for a less (T119M) and more (A19D and V30M) amyloidogenic variant of TTR. Moreover, we dissected the ΔΔG for each step of TTR tetramer dissociation by dividing the tetramer dissociation into AB and CD dimers (ΔΔG_d1_); the dissociation of these dimers into A, B, C and D monomers (ΔΔG_d2_) and the final denaturation of the separate monomers (ΔΔG_u_). It must be emphasized that denaturation experiments with urea or guanidine do not allow a direct assessment of each one of these ΔGs because the dissociation and denaturation steps significantly overlap. The scheme presented below summarizes the most affected steps in the dissociation-denaturation of each variant studied here. 

According to our analysis, there are eight newly discovered interactions involving residues Leu110 and Met119 in T119M, a structural change that might explain most of the increased thermodynamic stability of its tetramer. Additionally, these new interactions strengthened the contacts between the T4 hydrophobic nucleus and residues 15, 17, 108 and 110 of the T4-binding channels, inducing tighter binding of the hormone to T119M [38]. FoldX also predicted that, once the T119M monomers formed, they would present the same stability as A19D monomers because both are only slightly less stable than the WT-TTR monomers (~1 kcal/mol, Table 1). Our group has used high hydrostatic pressure to show that the T119M monomers do indeed present a stability comparable to that of the WT-TTR monomers [41]. In addition, because the partially unfolded monomers are the building blocks for TTR fibril formation, we envision that WT-TTR, T119M and A19D monomers would form amyloid fibrils with equal propensity. In fact, T119M monomers were able to form amyloid fibrils once they had similar kinetics as those of WT-TTR (unpublished results). Thus, the non-amyloidogenic character of T119M is explained by the enhanced stability of its tetramers; once monomers are formed, they will aggregate. Molecular dynamic simulations of the T119M tetramer support this view (Elena Papaleo, personal communication).

In the case of V30M, the tetramer and dimer are as stable as their WT counterparts. However, the unfolding step is significantly compromised in this variant (ΔΔG_u_ = 3.03 kcal/mol), and this decreased V30M monomer stability shifts the above equilibrium towards unfolded conformations, rendering this variant more amyloidogenic than the WT-TTR [20,23]. Interestingly, this prediction pointed in the same direction as the results obtained from chemical denaturation experiments performed with V30M. As shown before by Hurshman Babbes, Powers and Kelly (2008), the V30M monomers were destabilized by ~2.5 kcal/mol in relation to the WT-TTR monomers, and each tetramer presented a similar stability when Cm values were compared [23]. Thus, once V30M monomers are synthesized by the ribosome, their assembly into dimers and tetramers should be fast enough to avoid their aggregation into amyloids. 

In the case of A19D, the dissociation into dimers is favorable in relation to the WT-TTR by 4.65 kcal/mol, which, when added to the gently decreased stability of the A19D monomers (1.24 kcal/mol), would explain the enhanced amyloidogenicity of this variant. It must be noted that the patient is heterozygous (Figure 2) and so his tetramer population is composed of a mixture of WT and A19D subunits, likely displaying a gradient of stabilities ranging from a stable WT to highly destabilized A19D homotetramers. 

A structural analysis of the A19D model indicated a loss of several important interactions on the tetrameric interface relying on intermolecular contacts between residues Leu110 and Thr119, making A19D tetramer unstable (Figure 4B). Moreover, we observed a remarkable reorientation of the residues that are important for T4 hormone binding, namely Leu110 and Thr119. Because the drug Tafamidis was recently approved to treat FAP [42,43] [42], and other tetramer stabilizing compounds [44,45] use these pockets to bind to TTR to prevent its amyloidogenesis, our findings question the use of these compounds to treat this form of cardiac disease, as well as other diseases that involve structural changes in the T4 binding channels. It will be interesting to perform *in vitro* binding and aggregation inhibition assays as soon as the recombinant protein is available. 

**Figure 6:**
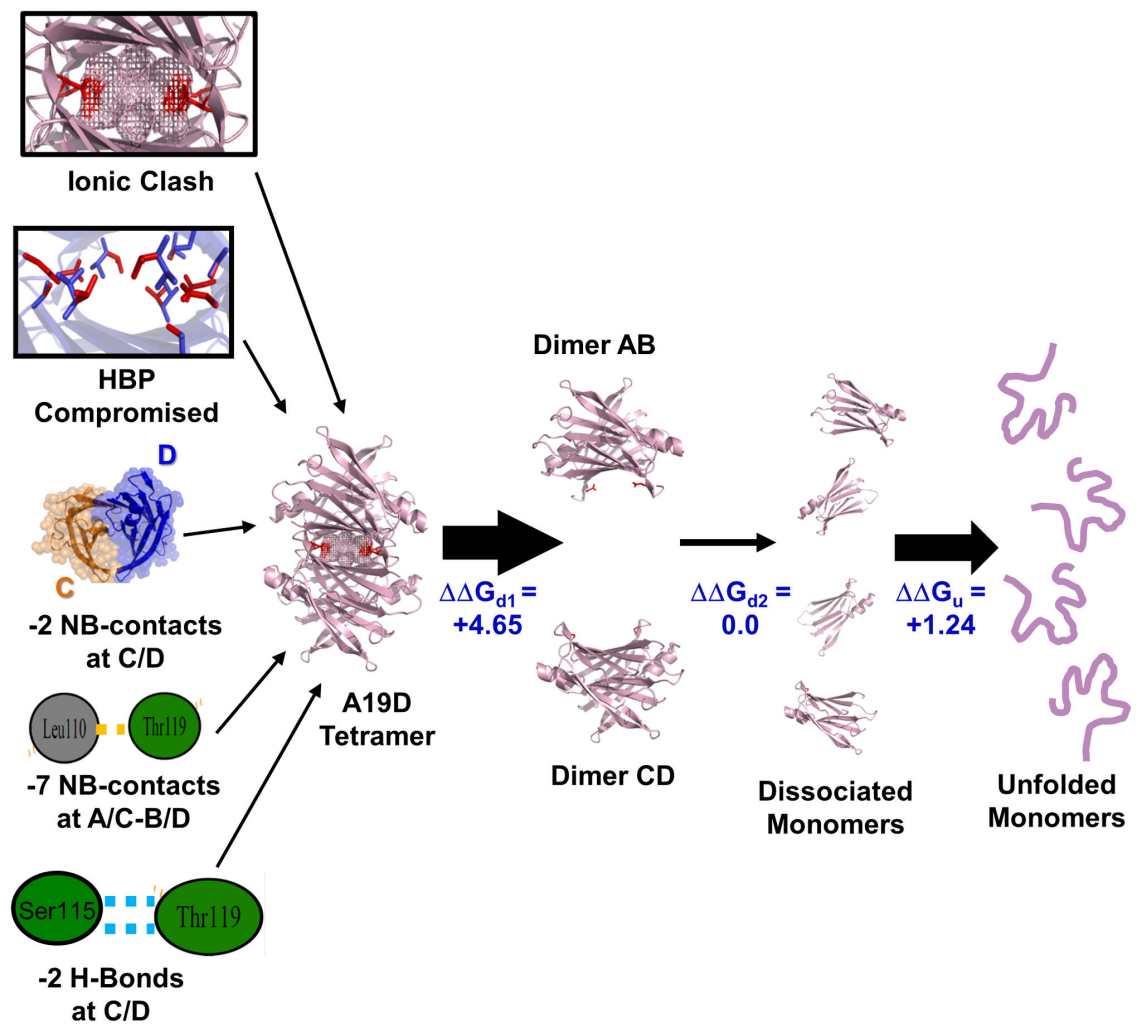
Summary of the A19D unfolding pathway highlighting the main structural changes identified here that might explain its decreased thermodynamic stability. All these changes, namely the concentration of four negative charges arising from Asp19 inside T4-binding channels 6Å apart (ionic clash), a complete reorientation of several important residues inside the T4-binding channels, the loss of seven non-bonded contacts between Leu110-Thr119 at the tetrameric interface, the loss of two H-bonds between Ser115-Thr119 and two non-bonded contacts, all at the C/D interface, were identified by FoldX and PDBsum.

**Figure 7:**
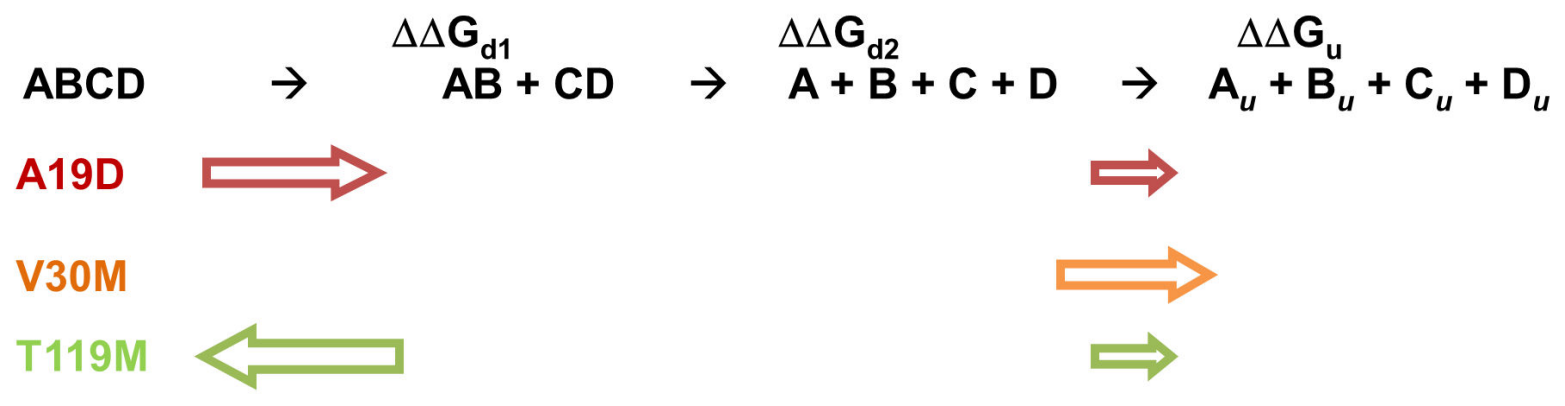
The tetramer-unfolded monomer equilibrium in A19D, V30M and T119M-TTR predicted by FoldX. The width and the direction of the colored arrows are related to the intensity and the side of reactions that the equilibrium would be shifted to, respectively. The most compromised step on the A19D pathway is the first step of dissociation (red arrow), formation of AB and CD dimers. Instead, V30M has the last step affected (orange arrow), unstable monomers. In the case of the non-amyloidogenic T119M, the equilibrium is shifted to the left, indicating no dissociation (green arrow).

The scheme presented in Figure 6 summarizes the unfolding pathway of A19D, highlighting the main structural changes identified here, which might explain its overall decreased thermodynamic stability, namely the concentration of four negative charges arising from Asp19 inside the T4-binding channels at 6Å apart (ionic clash), a complete reorientation of several important residues inside the T4-binding channels, the loss of seven non-bonded contacts between Leu110-Thr119 at the tetrameric interface, the loss of two H-bonds between Ser115-Thr119 and two non-bonded contacts, all at the C/D interface. Previous studies with engineered TTR tetramers have shown that the A/B and C/D interfaces are the strongest TTR tetramer interfaces, and A/C-B/D are the weakest ones [13,19]. The proposed mechanism for TTR dissociation assumes breakage at the *C*_*2*_ crystallographic tetramer axis (A/C-B/D interface) leading to the formation of dimers AB and CD, which then degrade into four monomers (Figure 6). Thus, it is possible that the loss of contacts present at the already weak A/C-B/D interface of A19D could shift the equilibrium towards tetramer dissociation. In addition to the number of contacts that were lost, some of them seem to be crucial for maintaining tetramer assembly, such as the one established between Leu110-Thr119, an interaction that is stronger in T119M (+8 new non-bonded contacts). Once the A/B and C/D dimers are formed, dimer C/D would still establish fewer interactions than its WT counterparts (-2 non-bonded contacts and -2 H-bonds, although Thr118-Ser115 makes a new H-bond, furnishing a total of -1 H-bonds at the C/D interface). The loss of these contacts does not seem to contribute to dimer stability, because the predicted ΔΔG_d2_ is zero. Overall, the tetramer-unfolded monomer equilibrium in A19D is shifted to the right (Figure 7), and considering that a partially unfolded monomer is the building block for fibril formation, this trend would lead this variant to form more amyloidogenic deposits in the heart. 

The recent CEPARM consolidation in our university hospital led to the identification of the rare A19D-TTR variant in a Brazilian patient, suggesting that other new, uncharacterized mutants could be identified in the coming years. We believe that FoldX could be used as a first tool to probe new TTR mutants’ stability and consequently amyloidogenicity. 

## Supporting Information

Figure S1 **Superimposition of the crystal structure of WT-TTR (blue) and the Foldx-generated model of A19D-TTR (red).** The main chains are shown as cartoons and side chains in blue lines and red sticks. The image was produced using PyMOL.
(PNG)

Table S1 **Number of non-bonded contacts involved in the dimer-dimer (A/C and B/D) interface in A19D, T119M and V30M in relation to the WT-TTR, highlighting the importance of the 110-119 pair.**
(DOCX)
