# Correction: Structure-Based Analysis of A19D, a Variant of Transthyretin Involved in Familial Amyloid Cardiomyopathy

**DOI:** 10.1371/annotation/2ea264c2-d70c-4a2e-b877-c084dc3a49db

**Published:** 2013-12-31

**Authors:** Priscila Ferreira, Oliveira Sant'Anna, Nathalia Varejão, Cinthia Lima, Shenia Novis, Renata V. Barbosa, Concy M. Caldeira, Franklin D. Rumjanek, Salvador Ventura, Marcia W. Cruz, Debora Foguel

The name of the second author is incorrect. The correct name is: Ricardo Sant'Anna. 

The correct Citation is: Ferreira P, Sant’Anna R, Varejão N, Lima C, Novis S, et al. (2013) Structure-Based Analysis of A19D, a Variant of Transthyretin Involved in Familial Amyloid Cardiomyopathy. PLoS ONE 8(12): e82484. doi:10.1371/journal.pone.0082484.

The correct abbreviation of the second author's name in the Author Contributions Statement is: RS.

