# Supplementary figures and images for: Structure-Based Analysis of A19D, a Variant of Transthyretin Involved in Familial Amyloid Cardiomyopathy
Source: PLoS One. 2013 Dec 17;8(12):e82484. doi: 10.1371/journal.pone.0082484 (PMC3866121; doi:10.1371/journal.pone.0082484)

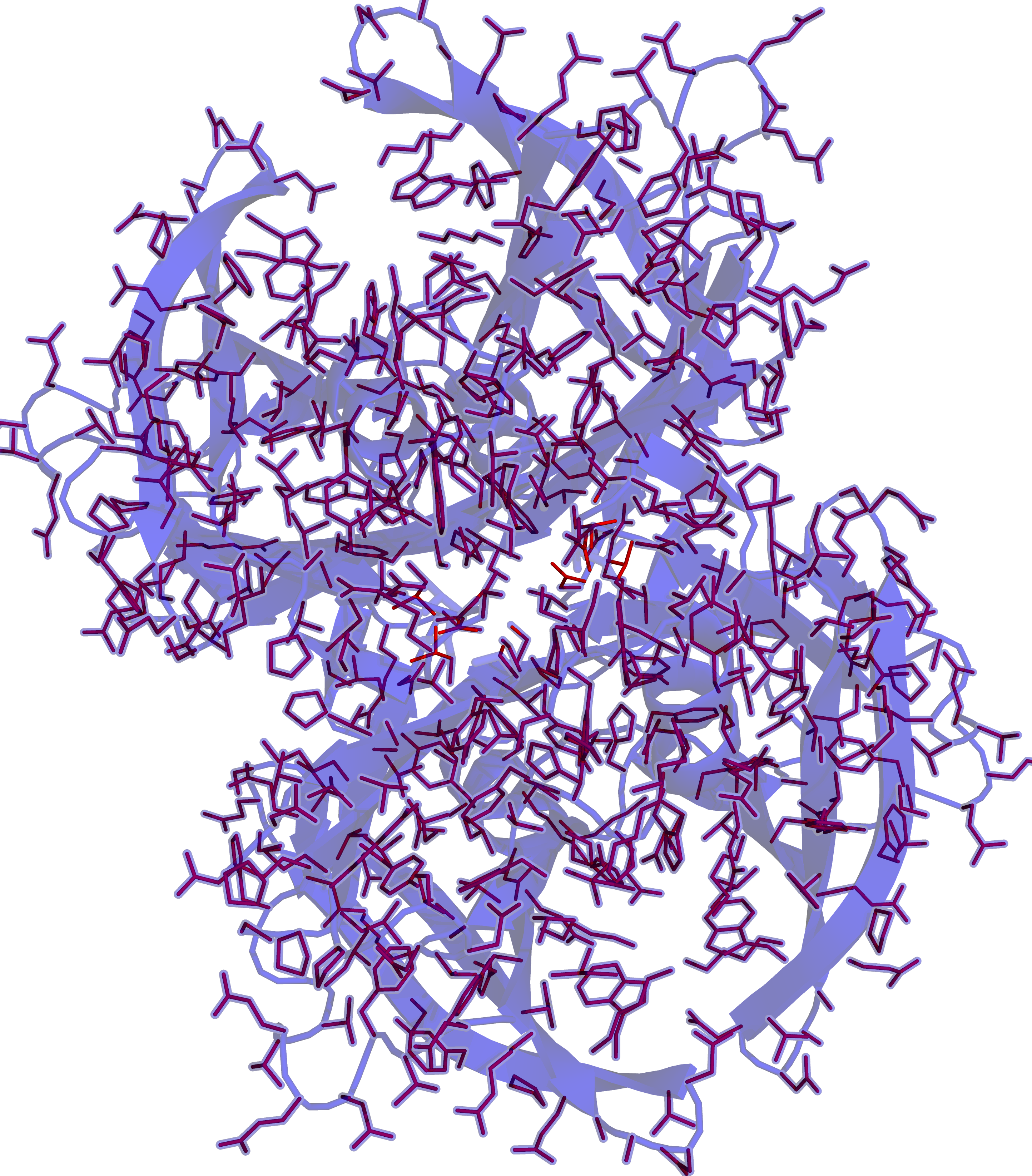

Supplement: Figure S1 — Superimposition of the crystal structure of WT-TTR (blue) and the Foldx-generated model of A19D-TTR (red). The main chains are shown as cartoons and side chains in blue lines and red sticks. The image was produced using PyMOL. (PNG) [file pone.0082484.s001.png]
