# Supplementary material for: Structure-Based Analysis of A19D, a Variant of Transthyretin Involved in Familial Amyloid Cardiomyopathy
Source: PLoS One. 2013 Dec 17;8(12):e82484. doi: 10.1371/journal.pone.0082484 (PMC3866121; doi:10.1371/journal.pone.0082484)
Supplement: Table S1 — Number of non-bonded contacts involved in the dimer-dimer (A/C and B/D) interface in A19D, T119M and V30M in relation to the WT-TTR, highlighting the importance of the 110-119 pair. (DOCX) [file pone.0082484.s002.docx]

| **Interface A/C** | **WT/A19D** | **WT/T119M** | **WT/V30M** |
| --- | --- | --- | --- |
| **A(D)19/T(M)119** | 0/1 | 0/0 | 0/0 |
| **L17/T(M)119** | 1/1 | 1/2 | 0/0 |
| **L17/V121** | 4/4 | 5/5 | 3/3 |
| **G22/A120** | 6/6 | 6/6 | 6/6 |
| **G22/V121** | 5/5 | 5/5 | 5/5 |
| **G22/V122** | 8/8 | 8/8 | 8/8 |
| **L110/T(M)119** | 8/3 | 5/13 | 6/6 |
| **Interface B/D** |  |  |  |
| **A(D)19/T(M)119** | 0/3 | 0/0 | 0/0 |
| **L17/T(M)119** | 0/0 | 0/1 | 0/0 |
| **L17/V121** | 3/3 | 3/3 | 3/3 |
| **G22/A120** | 6/6 | 6/6 | 6/6 |
| **G22/V121** | 4/4 | 4/4 | 4/4 |
| **G22/V122** | 9/9 | 9/9 | 9/9 |
| **P24/T123** | 1/1 | 1/1 | 1/1 |
| **L110/T(M)119** | 7/5 | 7/7 | 6/6 |

**Table S1. Number of non-bonded contacts enrolled in dimer-dimer (A/C and B/D) interface in A19D, T119M and V30M inrelation to the WT-TTR highlighting the importance of the pair 110-119.**
